# Comprehensive characterization and understanding of micro-fuel cells operating at high methanol concentrations

**DOI:** 10.3762/bjnano.6.203

**Published:** 2015-10-07

**Authors:** Aldo S Gago, Juan-Pablo Esquivel, Neus Sabaté, Joaquín Santander, Nicolas Alonso-Vante

**Affiliations:** 1 IC2MP, UMR-CNRS 7285, University of Poitiers, 4 rue Michel Brunet, 86022 Poitiers, France; 2 Instituto de Microelectrónica de Barcelona, IMB-CNM (CSIC), Campus UAB, 08193 Bellaterra, Barcelona, Spain

**Keywords:** fuel crossover, methanol, micro-fabrication, passive direct methanol fuel cell (DMFC), reversible hydrogen electrode (RHE)

## Abstract

We report on the analysis of the performance of each electrode of an air-breathing passive micro-direct methanol fuel cell (µDMFC) during polarization, stabilization and discharge, with CH_3_OH (2–20 M). A reference electrode with a microcapillary was used for separately measuring the anode the cathode potential. Information about the open circuit potential (OCP), the voltage and the mass transport related phenomena are available. Using 2 M CH_3_OH, the anode showed mass transport problems. With 4 and 6 M CH_3_OH both electrodes experience this situation, whereas with 10 and 20 M CH_3_OH the issue is attributed to the cathode. The stabilization and fuel consumption time depends mainly on the cathode performance, which is very sensitive to fuel crossover. The exposure to 20 M CH_3_OH produced a loss in performance of more than 75% of the highest power density (16.3 mW·cm^−2^).

## Introduction

In recent years micro-fabricated fuel cells such as passive micro-direct methanol fuel cells (µDMFC) have been proposed as promising systems for powering portable devices [1–3]. Due to the fuel crossover from the anode to the cathode [4–6], these systems have to use the fuel in very dilute concentrations (<4 M) [7]. Otherwise, a severe performance loss occurs [8–10]. Esquivel et al. have reported a highly performant and efficient passive micro-fuel cell [11–14]. Recently, the use of this device as an integrated power source and micro-pump for Lab-on-Chip applications [15] as well as low-cost paper-based micro-fuel cells [16] has been demonstrated. Some of the new research trends for such devices involve the development of new material structures for electrode and catalyst integration [17], especially for alkaline media and the focus on a micro-fabrication oriented scheme [18].

Due to the reduced dimensions (miniaturization) of the fuel cell components and the passive operation, the access of reactants to the catalytic sites in this micro-fuel cell is limited. Moreover, CO_2_ bubbles and formation of H_2_O contribute to this difficulty. While this problem can be partially overcome with an optimal design of micro-fabricated current collectors [11], the effects of fuel crossover and mass transport in this micro-system are still not clear [12–13].

The use of a reference hydrogen electrode (RHE) allows one to studying the fuel crossover and the performance of the anode and the cathode, separately [19–21]. Unfortunately, this method requires the modification of the electrode compartments. On the other hand, through a Luggin capillary placed closely to one of the electrodes it is possible to measure the performance of the anode and the cathode in proton exchange membrane (PEM) hydrogen fuel cells [22] and electrolysers [23]. However, this method has not been yet employed for studying the fuel crossover in a passive μDMF. In this work we study, in a simple and straightforward way, the performance of each electrode of a passive, air-breathing µDMFC, submitted to CH_3_OH up to 20 M. The stabilization and fuel consumption in the micro-system are also discussed.

## Results and Discussion

### *E*–*I* characteristics of the µDMFC

The Luggin capillary was placed directly on top of the gold-plated current collector of the anode in order to measure the anode and cathode potential separately. Several locations within the small volume of the fuel reservoir were tested. The position closest to the anode catalyst layer being the most effective. Placing the RHE at the cathode did not allow any measurement of electrode potentials, as the µDMFC is air-breathing and no liquid contact was established. Table 1 summarizes the cathode (*E*_cat_) and anode (*E*_ano_) potential, maximum current (*j*_max_) and power (*P*_max_) density of the µDMFC with different concentrations of CH_3_OH.

**Table 1:** Cathode (*E*_cat_) and anode (*E*_ano_) potential, maximum current (*j*_max_) and power (*P*_max_) density of the µDMFC with different concentrations of CH_3_OH.

*c*_MeOH_ / M	*E*_cat_ / V vs RHE	*E*_ano_ / V vs RHE	*j*_max_ / mA·cm^−2^	*P*_max_ / mW·cm^−2^

2	0.88	0.43	93.1	13
4	0.82	0.41	148.3	16.3
6	0.75	0.42	165.5	13.6
10	0.69	0.41	106.9	11.3
20	0.6	0.4	62.1	4

The Pt–Ru-based anode is the most active binary electrocatalyst for DMFCs. The high activity of Pt–Ru for methanol oxidation has been attributed to both a bifunctional mechanism [24] and a ligand (electronic) effect [25]. The bifunctional mechanism of Pt and Ru involves the adsorption of oxygen containing species on Ru atoms at lower potentials, promoting the oxidation of CO to CO_2_ on Pt [24]:

[1]
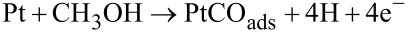


[2]
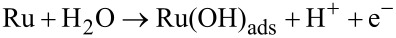


[3]
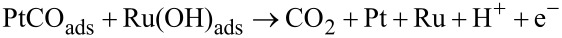


**Figure 1:**
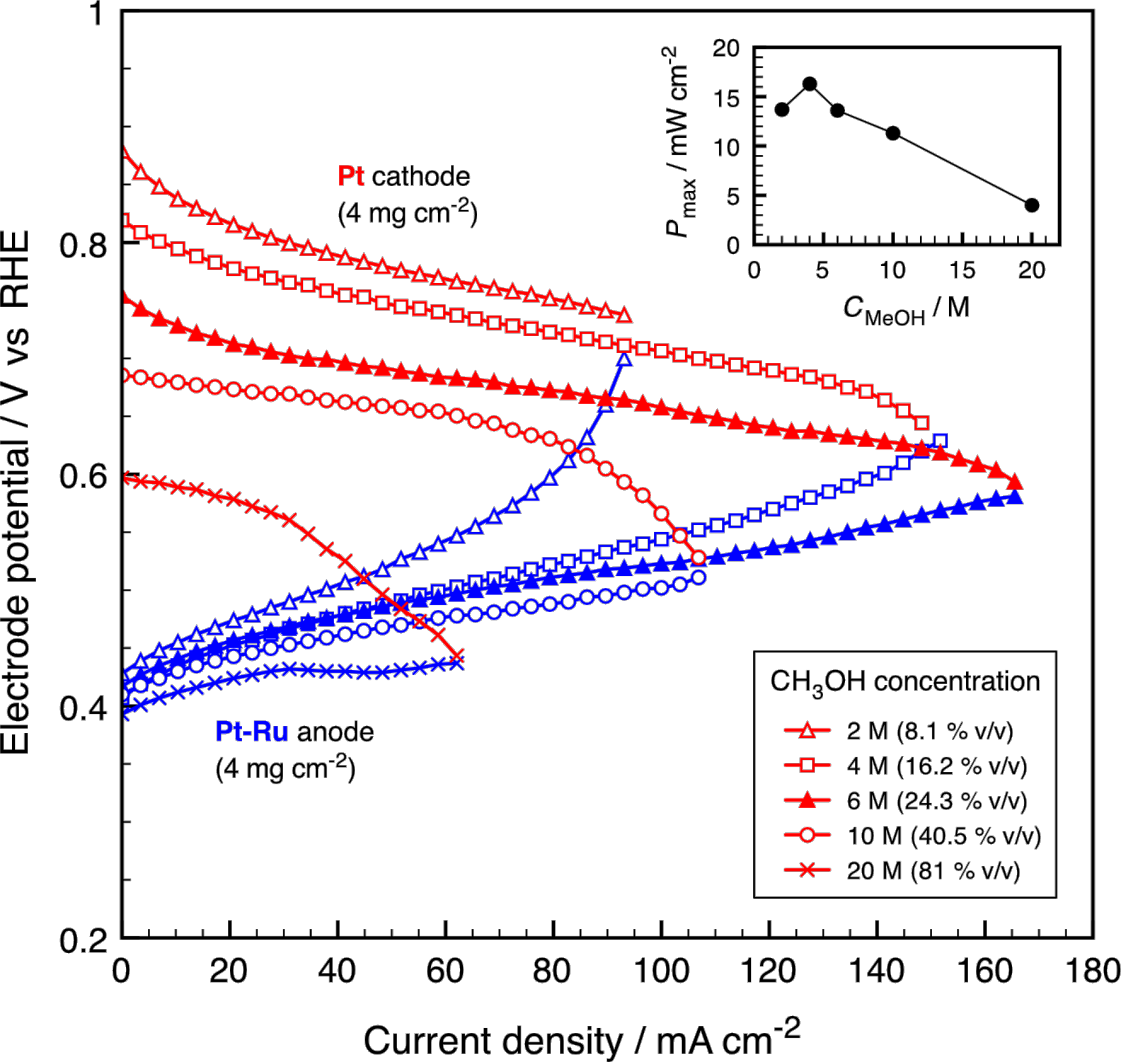
Current–potential characteristics of an air-breathing passive µDMFC. Pt–Ru anode (4 mg·cm^−2^), and Pt cathode (4 mg·cm^−2^). Inset shows the maximum power density as a function of the CH_3_OH concentration.

The electro-catalytic activity of the Pt–Ru catalyst depends on composition, structure, morphology, particle size and alloying degree [26–27]. On the other hand, the kinetics of CH_3_OH electro-oxidation on Pt–Ru depend strongly on temperature [28–29] and fuel concentration [30], and have a strong influence on the performance of the DMFC [8,20]. As shown in Figure 1, with 2 M CH_3_OH the anode of the µDMFC starts to depolarize quickly at ca. 50 mA·cm^−2^ reaching 0 V at 110 mA·cm^−2^. In normal size DMFC this depolarization occurs at current densities up to 400 mA·cm^−2^ depending on temperature, electrode and gas diffusion layer (GDL) design [20,31]. However, in our case the CH_3_OH transport of the µDMFC is controlled by the different open ratio of the anode current collector [11].

With 4 and 6 M CH_3_OH both electrodes contribute to this negative effect. With 10 and 20 M CH_3_OH the mass transport issues are inverted with the cathode being responsible for the low performance. The latter is flooded by the fuel that crosses through the membrane limiting the accessibility of O_2_ to the active Pt sites. The potential at the cathode, at a given current density, decreases gradually, 0.88 to 0.6 V vs RHE, as the fuel concentration increases. The reason is that the fuel crossover effect (mixed-potential developed between the oxygen reduction reaction (ORR) and the methanol oxidation reaction (MOR)) is more important [31].

The methanol concentration has a strong influence on the polarization curve of the anode, but it is mainly due to the small open ratio (23%) of the metallized silicon anode current collector produced by deep reactive ion etching (DRIE). It is designed in such a way that it reduces methanol crossover at low fuel concentrations. Using 20 M CH_3_OH causes a loss in performance in the passive µDMFC of 75% as compared to 4 M. It is thus clear that this system suffers from two problems: the Nafion^®^ in the membrane electrode assembly (MEA), which is permeable to the fuel and the lack of tolerance of the cathode catalyst.

### Stabilization

**Figure 2:**
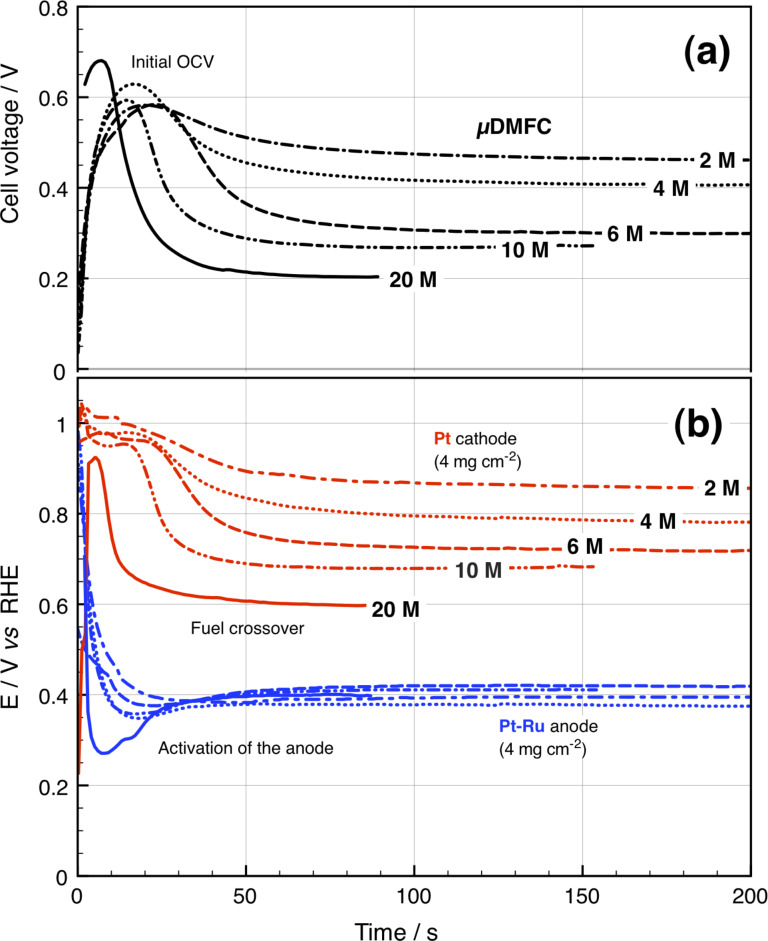
(a) Cell voltage and (b) electrode potential stabilization as 100 µL of CH_3_OH at a given concentration, was added into the fuel reservoir.

In addition to the current–potential curves, the stabilization and fuel consumption in the passive µDMFC was obtained versus the RHE (Figure 2). As the concentration of fuel increases, the µDMFC takes less time to stabilize. This condition mainly depends on the time the cathode electrode catalyst takes to get flooded and the catalyst to get eventually poisoned by the fuel that diffuses through the Nafion^®^ membrane of the MEA, the so-called “fuel crossover” effect [32]. The highest open circuit voltage (OCV) is attained only a few seconds after the fuel is added. The cathode potential reaches ca. 1 V vs RHE, which corresponds to the (ORR) onset potential on the nanostructured Pt in acidic media. Over time, a mixed-potential is generated by the oxidation of CH_3_OH at the cathode, which negatively affects the voltage of the cell. The anode potential remained practically at 0.4 V vs RHE and only varied slightly with the increase of concentration of the fuel.

The maximum open circuit voltage (OCV) was achieved using 20 M CH_3_OH since the anode got highly activated by the presence of a large amount of alcohol molecules. Nonetheless, it lasted only 7.1 s as the cathode potential decreased rapidly due to the fuel crossover in the micro-fuel cell. In overall, the time to attain equilibrium at the µDMFC decreased when increasing the fuel concentration as it occurs in a DMFC [32]. However, the result contrasts with that reported by Kho et al. [10] where the OCV always decreases when increasing the CH_3_OH concentration (<5 M) in DMFC.

**Figure 3:**
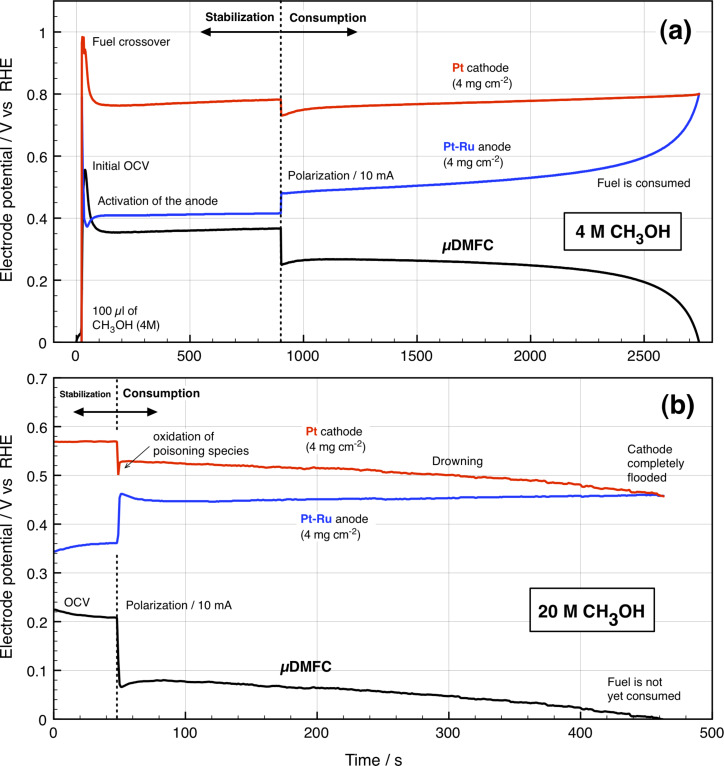
Cell and electrode potential measurements during the consumption of 100 µL of (a) 4 M and (b) 20 M CH_3_OH.

The evolution of µDMFC and electrode potential during the consumption of 4 M CH_3_OH is shown in Figure 3a. Once stabilized at an OCV of 0.37 V, the electronic load is connected, operating the cell at a constant current of 10 mA, delivering a cell voltage of 0.25 V. The output voltage remained practically constant for ca. 1140 s, decreasing 20% over the course of another 1380 s of operation. Finally, it goes sharply to 0 V in 240 s, indicating partial fuel consumption. By measuring the electrode potential separately, one can show that once the fuel consumption starts; the anode potential decreases by 70 mV and the cathode potential increases by 50 mV. The evolution of the anode potential is in good agreement with the evolution of the overall cell voltage. It confirms the depletion of CH_3_OH molecules at the surface of the Pt–Ru catalyst, which causes the drop in the cell voltage. The cathode potential was constant since the ORR occurs on the surface of the Pt catalyst and the oxidant (O_2_ from the air) is never depleted. During the fuel consumption it was observed that some CO_2_ bubbles randomly came out from the grid in the silicon current collector. This effect has been used to integrate this µDMFC in a microfluidic platform as a high performance micro-pump [15].

In contrast, using highly concentrated fuel (20 M) produced a completely different result, see Figure 3b. As the cell is polarized, the cathode potential quickly drops by 70 mV, and then it increases by 28 mV in less than 2 s, followed by a gradual decrease. This can be attributed to a brief oxidization of CH_3_OH species at the Pt surface to achieve equilibrium for the mixed-reaction: ORR and MOR. However, large amount of fuel crosses from the anode to the cathode, flooding and drowning it in the alcohol (observed with the naked eye). Therefore, oxygen is not further supplied at the Pt active sites and MOR at the cathode prevails over the ORR. The final result is a slow dropping in the cathode potential until the cell reaches 0 V making the µDMFC operable just for 420 s.

A quantitative evaluation of the efficiency of fuel cells can be assessed taking into account the faradaic and energy efficiency concepts [33]. A simple finite element model was built for this application, tuned and validated by comparing the results against experimental polarization curves and efficiencies. The faradaic efficiency allows evaluating the percentage of the theoretical fuel capacity that is actually being converted to current and it is computed by the following expression:

[4]
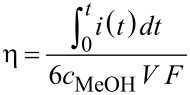


where *t* is the operating time, *i*(*t*) the measured current, *c*_MeOH_ the methanol concentration, *V* the solution volume, and *F* the Faraday constant.

**Figure 4:**
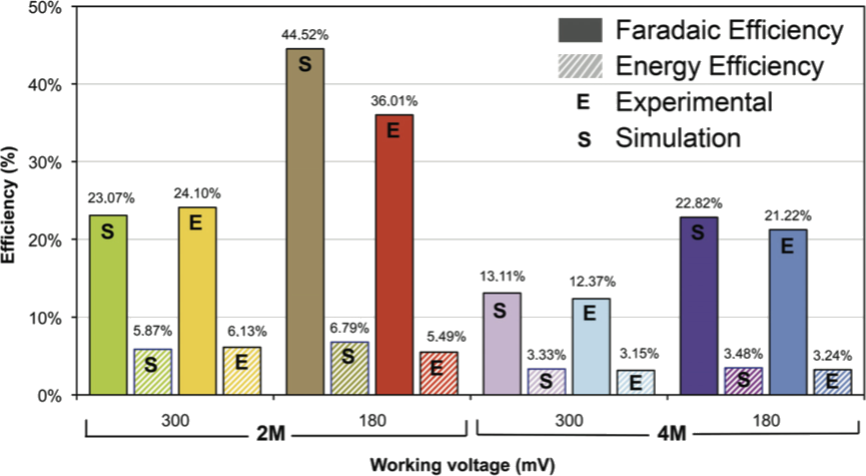
Comparison between experimental and modelled faradaic efficiency and energy efficiency of the micro-fuel cell fuel at different concentrations and operating voltages.

The energy efficiency of the fuel cell can be evaluated by comparing the power delivered by the cell when it is discharged to the energy that the fuel cell could deliver ideally, that is, when working at its theoretical voltage [33]:

[5]
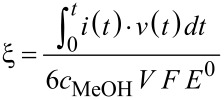


where *v*(*t*) is the operating voltage, *i*(*t*) the measured current and *t* the discharging time, *c*_MeOH_ the methanol concentration, *V* the solution volume and *F* the Faraday constant, and *E*^0^ = 1.18 V the theoretical cell voltage at 25 °C [8]. By simulating the discharge curve, the faradaic efficiencies can be obtained based on the previous equations. Figure 4 shows the comparison of experimental and simulated values of faradaic efficiency and energy efficiency obtained for 2 and 4 M at 180 and 300 mV. As it can be seen, the difference between the measured and calculated efficiencies is less than 2% in most cases, which supports the validity of the model and enable the prediction of the micro-fuel cell performance over time.

From the experiments, the energy efficiency is ca. 3.2% for 4 M CH_3_OH, dropping to 0.02% when using 20 M (not shown). This significant decrease in performance is entirely due to poisoning and flooding of the cathode. The methods and analyses reported in this work become relevant for improving the design and performance of passive micro-fuel cells. Without the use of a reference electrode, the conclusions previously discussed could only be speculated.

## Conclusion

The electrode performance of an air-breathing passive micro-direct methanol fuel cell (µDMFC) was studied. In this work we have focussed exclusively on studying a fuel cell operating with fuel concentrations up to 20 M, with a RHE that does not imply any modification to the cell architecture and no salt bridge is used. A maximum power density of 16.3 mW·cm^−2^ was attained with 4 M CH_3_OH, however it decreased by 85% when using almost pure methanol (20 M). With low concentrations of CH_3_OH (2 M), the anode showed mass transport issues, whereas with high concentrations (10 and 20 M CH_3_OH) the cathode failed, due to the fuel crossover. The maximum open circuit voltage (OCV) was achieved when adding 20 M CH_3_OH, but it lasted only 7.1 s as the fuel quickly poisoned the cathode. These observations were acquired by means of an RHE with a micro-capillary. It is simple and can be equally used for studying the performance of the anode and the cathode of other small fuel cells such as alkaline, microfluidic or bio-fuel cells. Thus, the design and micro-fabrication of parts and components such as current collectors, fuel reservoir, gas diffusion layers (GDL), the electrolyte membrane, and of course, the electrode catalysts, can be improved. The applied characterization procedure used in this work is expected to be useful for the development of future micro-fuel cells. In the case of such compact and miniaturized devices, the presented technique can help to understand the role of the different integrated components of micro-fuel cells.

## Experimental

**Figure 5:**
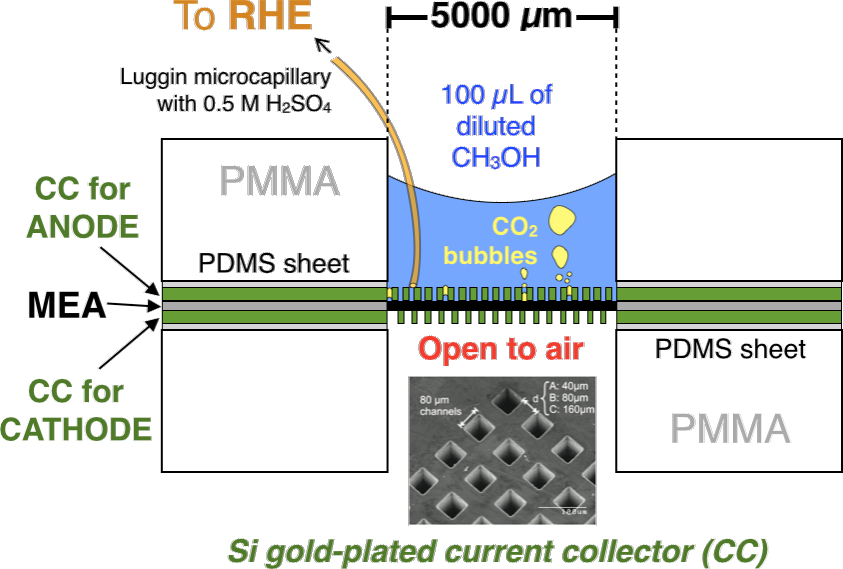
Scheme of the passive µDMFC with Luggin micro-capillary.

More details on the fabrication of the passive micro-direct methanol fuel cell (µDMFC) are given in [11,14]. In short, the micro-fuel cell possessed two Si-gold plated current collectors made by deep reactive ion etching process (DRIE), with optimized open area geometries. Metallized silicon current collectors with 23 and 40% open ratio were chosen for the anode and cathode, respectively. The silicon plates act as current collectors and gas diffusion layer (GDL) delivering the reactants to the catalyst layers that reach both sides of the membrane electrode assembly (MEA) by diffusion. A small piece of commercial MEA (E-TEK^®^) was put between the Si current collectors, leaving an exposed active area of 0.29 mm^2^. According to the provider the catalysts mass loading of the anode Pt–Ru/C, and at that of the cathode Pt/C is 4 mg·cm^−2^, and Nafion^®^ 117 is used as membrane. The fuel reservoir was on top of the anode. As fuel, 100 µL of CH_3_OH (Sigma-Aldrich), with concentrations of 2, 4, 6, 10 and 20 M, was added in the reservoir every time a measurement was carried out. A Keithley 2400 SourceMeter^®^ served as an electronic load and the current and voltage produced by the fuel cell were measured. Cathode and anode electrode potentials were measured using the reference hydrogen electrode (RHE) coupled to a Luggin micro-capillary plunged into the reservoir (Figure 5) both filled with 0.5 M H_2_SO_4_ (Sigma-Aldrich). All measurements were carried out at room temperature.

## References

[1] Achmad F, Kamarudin S K, Daud W R W, Majlan E H (2011). Appl Energy.

[2] Kundu A, Jang J H, Gil J H, Jung C R, Lee H R, Kim S-H, Ku B, Oh Y S (2007). J Power Sources.

[3] Pinochat T, Zhao T S (2009). MEMS-Based Micro Fuel Cells as Promising Power Sources for Portable Electronics. Micro fuel cells: principles and applications.

[4] Song S Q, Zhou W J, Li W Z, Sun G, Xin Q, Kontou S, Tsiakaras P (2004). Ionics.

[5] Scott K, Taama W M, Argyropoulos P, Sundmacher K (1999). J Power Sources.

[6] Lai Q-Z, Yin G-P, Wang Z-B, Du C-Y, Zuo P-J, Cheng X-Q (2008). Fuel Cells.

[7] Zhao T S, Yang W W, Chen R, Wu Q X (2010). J Power Sources.

[8] Liu J G, Zhao T S, Chen R, Wong C W (2005). Electrochem Commun.

[9] Xu C, Faghri A, Li X, Ward T (2010). Int J Hydrogen Energy.

[10] Kho B K, Bae B, Scibioh M A, Lee J, Ha H Y (2005). J Power Sources.

[11] Esquivel J P, Sabaté N, Santander J, Torres-Herrero N, Gràcia I, Ivanov P, Fonseca L, Cané C (2009). J Power Sources.

[12] Torres N, Santander J, Esquivel J P, Sabaté N, Figueras E, Ivanov P, Fonseca L, Grácia I, Cané C (2008). Sens Actuators, B.

[13] Sabate N, Esquivel J, Santander J, Torres N, Gracia I, Ivanov P, Fonseca L, Figueras E, Cane C (2008). J New Mater Electrochem Syst.

[14] Esquivel J P, Sabaté N, Santander J, Torres N, Cané C (2008). Microsyst Technol.

[15] Esquivel J P, Castellarnau M, Senn T, Löchel B, Samitier J, Sabaté N (2012). Lab Chip.

[16] Esquivel J P, Del Campo F J, Gómez de la Fuente J L, Rojas S, Sabaté N (2014). Energy Environ Sci.

[17] Verjulio R W, Santander J, Sabaté N, Esquivel J P, Torres-Herrero N, Habrioux A, Alonso-Vante N (2014). Int J Hydrogen Energy.

[18] Sabaté N, Esquivel J P, Santander J, Hauer J G, Verjulio R W, Gràcia I, Salleras M, Calaza C, Figueras E, Cané C (2014). Microsyst Technol.

[19] Du C Y, Zhao T S, Yang W W (2007). Electrochim Acta.

[20] Kim Y J, Hong W H, Woo S I, Lee H K (2006). J Power Sources.

[21] Küver A, Vogel I, Vielstich W (1994). J Power Sources.

[22] Hinds G, Brightman E (2012). Electrochem Commun.

[23] Brightman E, Dodwell J, van Dijk N, Hinds G (2015). Electrochem Commun.

[24] Watanabe M, Motoo S (1975). J Electroanal Chem.

[25] Frelink T, Visscher W, van Veen J A R (1995). Surf Sci.

[26] Aricò A S, Srinivasan S, Antonucci V (2001). Fuel Cells.

[27] Tripkovic A V, Popovic K D, Grgur B N, Blizanac B, Ross P N, Markovic N M (2002). Electrochim Acta.

[28] Gasteiger H A, Markovic N, Ross P N, Cairns E J (1994). J Electrochem Soc.

[29] Chu D, Gilman S (1996). J Electrochem Soc.

[30] Khazova O A, Mikhailova A A, Skundin A M, Tuseeva E K, Havránek A, Wippermann K (2003). Fuel Cells.

[31] Ravikumar M K, Shukla A K (1996). J Electrochem Soc.

[32] Ramya K, Dhathathreyan K S (2003). J Electroanal Chem.

[33] Jiang R, Rong C, Chu D (2004). J Power Sources.

